# Gene Expression Patterns in Pancreatic Tumors, Cells and Tissues

**DOI:** 10.1371/journal.pone.0000323

**Published:** 2007-03-28

**Authors:** Anson W. Lowe, Mari Olsen, Ying Hao, Sum P. Lee, Kyu Taek Lee, Xin Chen, Matt van de Rijn, Patrick O. Brown

**Affiliations:** 1 Department of Medicine, Stanford University Medical Center, Stanford, California, United States of America; 2 Department of Biochemistry, Stanford University Medical Center, Stanford, California, United States of America; 3 Department of Pathology, Stanford University Medical Center, Stanford, California, United States of America; 4 Stanford Digestive Disease Center, Stanford University Medical Center, Stanford, California, United States of America; 5 Howard Hughes Medical Institute, Stanford University Medical Center, Stanford, California, United States of America; 6 Veterans Administration Puget Sound Health Care System, Seattle, Washington, United States of America; 7 Department of Medicine, Samsung Medical Center, Sungkyunkwan University School of Medicine, Seoul, Korea; 8 Department of Biopharmaceutical Sciences, University of California, San Francisco, California; South African National Bioinformatics Institute, South Africa

## Abstract

**Background:**

Cancers of the pancreas originate from both the endocrine and exocrine elements of the organ, and represent a major cause of cancer-related death. This study provides a comprehensive assessment of gene expression for pancreatic tumors, the normal pancreas, and nonneoplastic pancreatic disease.

**Methods/Results:**

DNA microarrays were used to assess the gene expression for surgically derived pancreatic adenocarcinomas, islet cell tumors, and mesenchymal tumors. The addition of normal pancreata, isolated islets, isolated pancreatic ducts, and pancreatic adenocarcinoma cell lines enhanced subsequent analysis by increasing the diversity in gene expression profiles obtained. Exocrine, endocrine, and mesenchymal tumors displayed unique gene expression profiles. Similarities in gene expression support the pancreatic duct as the origin of adenocarcinomas. In addition, genes highly expressed in other cancers and associated with specific signal transduction pathways were also found in pancreatic tumors.

**Conclusion:**

The scope of the present work was enhanced by the inclusion of publicly available datasets that encompass a wide spectrum of human tissues and enabled the identification of candidate genes that may serve diagnostic and therapeutic goals.

## Introduction

The aim of this study is to provide a global assessment of gene expression patterns for the major neoplastic diseases of the pancreas. We demonstrate that the inclusion of a diverse source of diseased and normal pancreatic tissues for analysis using DNA microarrays enhances the identification of gene expression patterns that can be attributed to specific cell types and pancreatic diseases.

Pancreatic adenocarcinoma represents a significant health burden in industrialized countries and represents one of the most lethal human cancers. It is the fourth-leading cause of cancer-related death in the United States with a five-year survival rate of 4% [1]. Oligonucleotide microarrays, cDNA microarrays, and serial analysis of gene expression technologies have been used to characterize the gene expression profile of pancreatic adenocarcinomas [2]–[5]. These studies have identified candidate genes that may play a role in disease pathogenesis or may serve as clinical markers of disease. The analysis of gene expression data for pancreatic cancer, however, is problematic because of the pronounced desmoplasia associated with the disease. The actual neoplastic cells often comprise a minority of the total cell population within pancreatic tumors, with the majority belonging to stromal elements. When pancreatic adenocarcinomas have been compared to normal pancreatic tissues, the robust desmoplastic response produced by stromal cells contributes to a gene expression profile that may dominate over that contributed by the neoplastic cells themselves. Because other pancreatic diseases are also known to result in significant desmoplasia, further confounding the interpretation of the patterns seen in cancers, this study approached the problem by expanding the tissues examined to include a wide variety of diseased and normal pancreatic tissues. The present study used samples derived from pancreatic tumors of exocrine, endocrine, and mesenchymal origin. In addition, isolated pancreatic islets and ducts from normal tissues as well as pancreatic cancer cell lines were used to enhance analysis of the gene expression profile. Each pancreatic tissue and cell line contributes to an interpretative framework that facilitates identification of genes whose expression is most characteristic of specific cell types and diseases. For example, genes that participate in the desmoplastic response can be distinguished because their expression is a shared feature in a variety of different pancreatic diseases in which this response is seen.

Islet cell tumors represent a second major pancreatic tumor whose pathogenesis is poorly understood and for which effective therapies are lacking. In contrast to previously published studies that compared gene expression between islet tumors and normal islets, the current study also examines gene expression in islet tumors in the context of the whole pancreas and other pancreatic diseases [6]–[8].

The expanding collection of publicly accessible gene expression data also provides an opportunity to compare and enhance the dataset provided by this study. For example, the gene expression profiles of a wide spectrum of normal human tissues [9] provide an opportunity to compare gene expression profiles of pancreatic cancer to a detailed overview of the cells and tissues of the human body, and thereby focus on those features most likely to provide diagnostic markers for detection of disease.

## Materials and Methods

### Samples and RNA isolation

All participating patients provided consent prior to surgery. The resected tissue was immediately frozen in liquid nitrogen, and stored at −80°C. A portion of the sample was processed for histopathology. Normal pancreatic islets and pancreatic ducts were obtained from organ donors. Samples were obtained with approved human subjects protocols from Stanford University, Samsung Medical Center (Seoul, South Korea), University of Washington, and the Cooperative Human Tissue Network. Data derived from 11 adenocarcinoma samples and 5 non-tumor pancreatic samples included in this report were previously published in collaboration with Johns Hopkins University [5]. The present study represents the addition of 91 microarray studies that includes additional adenocarcinomas, islet cell tumors, other non-adenocarcinomas, benign pancreatic disease, normal isolated pancreatic islets and ducts, and pancreatic cancer cell lines.

Pancreatic cancer cell lines were obtained from the American Type Culture Collection (Manassas, VA) and included AsPC-1 (#CRL-1682), BxPC-3 (#CRL-1687), Capan-1 (#HTB-79), Capan-2 (#HTB-80), CFPAC-1 (#CRL-1918), Hs 766T (#HTB-134), MIA PaCa-2 (#CRL-1420), PANC-1 (#CRL-1469), SU.86.86 (#CRL-1837). The cells were grown to 80–90% confluence in media specified for each cell type. Islets were isolated from pancreata as previously described [10]. Cells derived from human pancreatic ducts were initially expanded as a primary culture and only early passage cells that retained responsiveness to secretin stimulation were used [11].

Total RNA for all samples was extracted using Trizol (Invitrogen, Carlsbad, CA) followed by mRNA isolation using FastTrack^®^ mRNA (Invitrogen, Carlsbad, CA). RNA amplification was performed for the isolated pancreatic duct cells and islets, 3 adenocarcinomas, and the reference mRNA standards using the procedure described by Wang et al. [12]. Two rounds of amplifications were used.

### Microarray Procedure

DNA microarrays containing 41,125 clones, representing approximately 24,473 unique genes were printed on microscope slides and processed for hybridization as previously described (http://cmgm.stanford.edu/pbrown/protocols/index.html) [13], [14]. The sample mRNA was labeled with Cy5-UDP and a common reference RNA derived from a panel of cell lines was labeled with Cy3-UDP (Amersham Biosciences, Piscataway, NJ) [14]. The arrays were scanned using a GenePix 4000A scanner (Axon Instruments, Molecular Devices Corp., Palo Alto, CA).

Features containing artifacts or blemishes were flagged for exclusion from further analysis using GenePix Pro 5.0 (Axon Instruments). The raw data was deposited in the Stanford Microarray Database (http://genome-www5.stanford.edu) [15]. A total of 9,259 unique genes (represented by 16,959 spots) satisfied subsequent filtering by exhibiting a mean signal intensity at least 1.5 fold greater than background for either the Cy5 or Cy3 channels. Only genes for which acceptable data were obtained in at least 70% of the samples and that showed at least a 1.5-fold difference in expression from the mean for at least 1 sample were retained. The expression data were visualized using Treeview [16]. Analysis included a hierarchical clustering algorithm that was applied to both the genes and arrays, and a supervised analysis by multi-class comparison using Significance Analysis of Microarrays (SAM) software [16], [17]. Identification of genes capable of discriminating between classes using shrunken centroids employed Prediction Analysis of Microarrays (PAM) software [18]. Gene annotations were obtained using SOURCE (http://source.stanford.edu) [19]. Genes predicted to encode secretory and membrane proteins were identified using the SignalIP software program that analyzes amino acid sequences for signal peptides (HMM/S mean score method)[20] or the TMHMM program that identifies transmembrane domains[21]. Additional putative secretory and membrane proteins were identified by interrogating an empirically-derived dataset[22]. Differentially expressed genes in pancreatic adenocarcinoma that are associated with areas of chromosome gains or loss were identified using comparative genomic hybridization data of the 6 cell lines used in the present study[23]. A high resolution map for regions of DNA copy number gains and losses was obtained using the CLuster Along Chromosome (CLAC) method[24].

The entire primary dataset is available at the Stanford Microarray Database (http://genome-www5.stanford.edu/) or the Lowe laboratory website (http://www.stanford.edu/group/lowelab/)

### Immunohistochemistry and In-situ hybridization

Immunohistochemistry was performed using Dako Envision Plus (Glostrup, Denmark) following the manufacturer's instruction. Rabbit anti-axin2 serum was from Zymed (Invitrogen, Carlsbad, CA). Tissue microarrays constructed to evaluate axin2 expression contained 13 normal pancreata and 26 pancreatic adenocarcinomas. Rabbit antibodies against LGALS4 were produced against peptide sequence NH2-PPYPGPGHCHQQLNS, and for MUC13 peptide sequence NH2-DPEEKHSMAYQDLHSEC (AnaSpec, Inc., San Jose, CA).

*In situ* hybridization was performed as previously described using paraffin-embedded sections [25].

## RESULTS

Gene expression profiles were obtained using cDNA microarrays for a total of 80 surgically removed pancreatic tissue samples (Table 1). The majority of the tumors removed were determined by pathology to be adenocarcinomas. Other pancreatic tissues collected included islet cell tumors, rare pancreatic tumors, chronic pancreatitis, and cystic disease. When available, non-tumor tissue was also collected. Normal pancreatic tissue was obtained from 9 organ donors.

**Table 1:** Samples used in the study

**Adenocarcinoma** *	**43**	
**Islet cell tumors (4 patients)**	6	
**Other tumors**	**4**	
osteoclast giant cell tumor (1 patient)		2
liposarcoma		1
lymphoma		1
**Benign disease**	**5**	
chronic pancreatitis		3
pseudocyst		1
mucinous cyst		1
**Non-tumor and normals**	**23**	
**Pancreatic cancer cell lines**	**9**	
**Primary pancreatic ducts**	**5**	
**Primary pancreatic islets**	**9**	

* 3 adenocarciinomas were used solely as amplified samples.

Additional samples included primary cultures of human pancreatic duct cells, purified human islets from normal donors, and nine commercially available pancreatic cancer cell lines. The human pancreatic duct cells and human islets were harvested from 5 and 9 different donors, respectively, and were processed separately and not pooled. RNA derived from the pancreatic duct cells and purified islets required antisense RNA amplification for the microarray analysis because of lower amounts of starting material.

**Figure 1:**
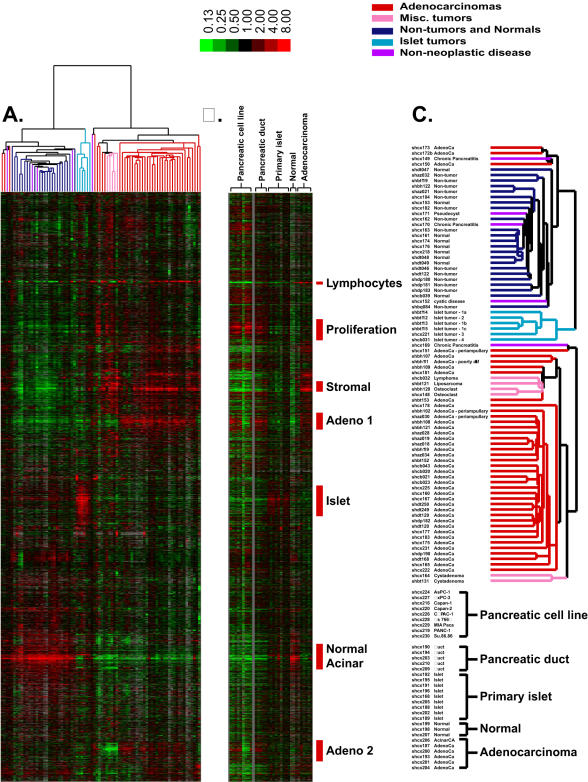
Unsupervised hierarchical clustering of the patterns of variation in expression for 9,259 genes (represented by 16,959 cDNA) in 80 pancreatic tissue specimens (A). The image uses a color code to represent relative expression levels. Red represents expression levels greater than the mean for a given gene across all samples. Green represents expression levels less than the mean across samples. A color bar (*top*) relates color code to the magnitude of the differences in gene expression relative to the all-sample means for each gene. Grey indicates missing or excluded data. (B) Image representing gene expression in pancreatic cell lines, primary pancreatic duct and islet cells, normal pancreas, and adenocarcinomas. The cell lines and amplified samples were each mean-centered separately. The genes presented are organized and displayed in an identical manner to the genes shown in panel (A). (C) Enlarged view of the array dendrogram shown in panel A along with sample identification.

We first used an unsupervised hierarchical clustering approach to organize and explore the data and highlight groups of genes and groups of samples with similar gene expression patterns[16]. Based on similarities in their overall gene expression profiles, most samples were clustered in a manner consistent with their original pathologic classification, yielding distinct clusters of adenocarcinomas, islet cell tumors, and normal pancreatic tissues (Figure 1A). Thirty-seven of 40 adenocarcinoma samples were grouped in one of the two major branches of the clustering dendrogram, of which 31 shared very similar expression profiles and were clustered within the same node. Six adenocarcinomas clustered in an adjoining node with lymphoma and other mesenchymal tumors, including 3 adenocarcinomas (shbh111, shbh109, shbt153, see Data S1 for clinical details) with atypical pathologic features. These adenocarcinomas were distinguished by higher expression of genes associated with proliferation and lower expression of the genes in the “Adeno” clusters 1 and 2. Three adenocarcinomas that clustered away from the majority of adenocarcinomas (shcx173, shcx172b, and shcx150) were distinguished by significant expression of genes encoding digestive enzymes, such as pancreatic amylase (AMY2A) and elastase (ELA3A), suggesting the presence of normal acinar cells in addition to the neoplastic component. The gene expression profiles for most of the adenocarcinomas were distinct from non-neoplastic tissues as well as other pancreatic tumors, including lymphomas, osteoclast tumors, liposarcomas, and islet-cell tumors. Among the adenocarcinomas, no significant segregation was observed on this analysis that would suggest subclassification into distinct molecular subtypes that might be associated with differences in tumor or cellular staging criteria. Clinical outcomes following surgery were known for 16/39 adenocarcinomas in this dataset (Data S1). The disease-free interval for almost all patients was less than 2 years. Among these samples, there was no indication that hierarchical clustering resulted in any classification by survival length.

Islet cell tumors were derived from four patients. Three samples from one patient representing separate metastatic lesions are included. Although immunohistochemistry revealed different secretory products for 3 patients, the tumors shared sufficient similarity in gene expression profiles to be clustered together.

Other pancreatic tumors that did not cluster with the adenocarcinomas or islet cell tumors included a pancreatic sarcoma, lymphoma, and osteoclast-like giant cell tumor. Although only single cases were represented for these rare tumors, they clustered together with the major common feature consisting of genes characteristic of the desmoplastic response, and relatively high expression of genes associated with proliferation.

Non-tumor and normal whole pancreatic tissues consistently clustered separately from the diseased tissues. The analysis did not distinguish between uninvolved non-tumor tissue from patients with cancer and pancreatic tissue from normal donors without any history of pancreatic disease.

Unsupervised hierarchical clustering also highlighted clusters of genes specifically associated with adenocarcinomas, islet cell tumors, and normal pancreata. The diversity of tumors and cell types in this sample set gave rise to a diversity of gene expression patterns, many of which appear to represent specific cell types. Defining the source of the distinctive gene expression patterns was facilitated by projecting the expression data for clonal pancreatic cancer cell lines, primary pancreatic duct cultures, and isolated pancreatic islets alongside the data derived from the previous clustering analysis (Figure 1B).

**Figure 2:**
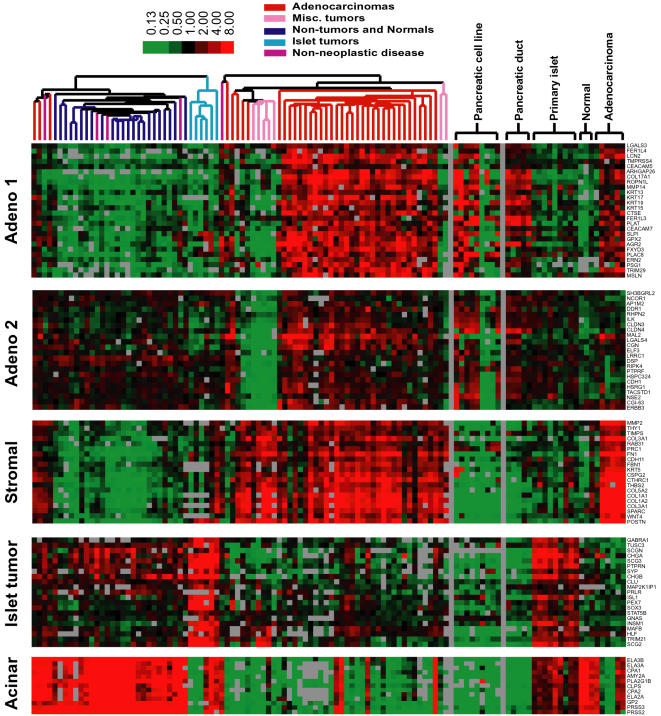
*Expanded view of gene clusters shown in* *Figure 1A*.Color code and experimental conditions are as described for Figure 1.

**Figure 3:**
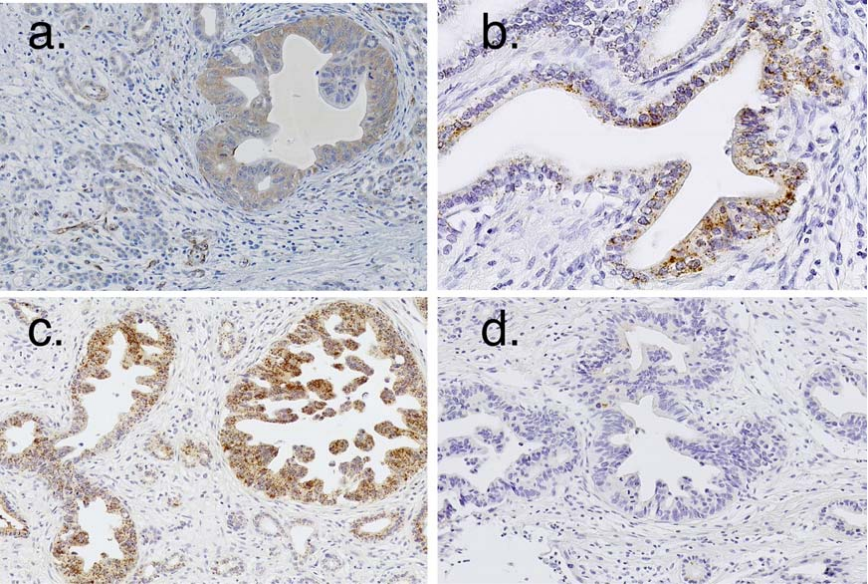
*Immunohistochemistry of paraffin embedded pancreatic adenocarcinomas.* Anti-galectin 4 (LGALS4) antibody (panel a). *In situ* hybridization for collagen 17A1 (COL17A1) (panel b) and mucin 13 (MUC13) (panel c). *In situ* hybridization sense control for MUC13 (panel d).

Of particular interest in this study was the segregation of genes associated with adenocarcinomas cells themselves from those involved in the desmoplastic response. Two gene clusters, labeled “Adeno 1” (297 genes) and “Adeno 2” (88 genes) (Figure 2), were characterized by higher expression in adenocarcinomas compared to other tumors or normal pancreatic tissues. The separation into 2 gene clusters is due to the higher relative expression of genes in adenocarcinomas compared to normal tissues in the “Adeno 1” cluster, as compared to the “Adeno 2” gene cluster. The specificity of genes within the adenocarcinoma clusters is supported by their lower expression in lymphoma, sarcoma, osteoclast-like cells, and islet cell tumors. Contained within each cluster are genes whose expression was previously associated with pancreatic adenocarcinoma, including keratin 19 (KRT19), anterior gradient 2 homolog (AGR2), v-erb-b2 avian erythroblastic leukemia viral oncogene homolog 3 (ERBB3), mesothelin (MSLN), and prostate stem cell antigen (PSCA) [5], [26]–[28]. Evidence that hierarchical clustering successfully distinguished between genes expressed predominantly in neoplastic epithelial cells and in stromal cells, respectively, is illustrated by two previously characterized matrix metalloproteinases, MMP14 and MMP2[25]. Expression of MMP14 by adenocarcinoma cells has been previously established by *in situ* hybridization, which is consistent with its clustering within the “Adeno 1” cluster. MMP2 is likewise associated with a separate cluster consistent with its expression by stromal cells (Figure 2). *In situ* hybridization or immunocytochemistry was performed for selected genes from each “Adeno” cluster and confirmed that the enhanced gene expression was indeed derived from the neoplastic cells (Figure 3).

We used significance analysis of microarrays (SAM), a supervised approach of analysis that incorporates an assessment of statistical significance, to identify genes that are differentially expressed in adenocarcinomas compared to the normal pancreas and other tumors in the dataset [17]. A total of 293 genes with an average expression at least 3-fold or higher in adenocarcinomas compared to non-adenocarcinoma samples were identified, with a false discovery rate of<.00001%. These genes were ranked based on the statistical significance of their differential expression in pancreatic adenocarcinoma versus other samples (Data S2). Among the 293 ranked genes, 176 are associated with the previously identified “Adeno 1” and “Adeno 2” clusters, and 87 genes are associated with the Stromal cluster. These genes include many not previously associated with pancreatic adenocarcinoma, such as galectin-4 (LGALS4), mucin 13 (MUC13), secretory leukocyte protease inhibitor (SLPI), collagen 17A1 (COL17A1), and ropporin 1-like (ROPN1L). Available annotations with respect to function and subcellular location revealed genes that function in signal transduction, the cytoskeleton, the extracellular matrix, and cellular metabolism (Data S2). Recent studies using comparative genomic hybridization of genomic DNA have produced a high resolution map for regions of DNA gains and losses for pancreatic cancer cell lines, including the 6 used in the present study. When used as a guide, 31 highly expressed genes and 26 genes expressed at lower levels in adenocarcinomas were also associated with corresponding areas of frequent DNA copy gains and losses (Data S3). Supporting the ductal origin of pancreatic adenocarcinomas, the majority of genes clustered in “Adeno 1” and “Adeno 2” were similarly highly expressed in isolated pancreatic duct cells. The same genes also showed higher expression in clonal pancreatic cancer cell lines. Among the commonly available adenocarcinoma cell lines, only a subset consisting of AsPC1, BXPC3, Capan-1, Capan-2, and CFPAC-1, consistently exhibited higher expression of genes that were similarly elevated in pancreatic adenocarcinomas (Figure 2). In contrast, genes in the stromal cluster exhibited much lower expression in the pancreatic adenocarcinoma cell lines and primary pancreatic duct cells. Significance analysis was also used to identify genes differentially expressed between primary duct and acinar cells. Because acinar cells comprise ∼90% of the pancreas, differences in gene expression between pancreatic duct and acinar cells were determined by comparing expression patterns of normal isolated pancreatic ducts with that of the normal whole pancreas derived from organ donors. A total of 1,476 genes with at least a 3-fold difference in expression between the two classes were identified with a false discovery rate of 0.05%. Again, genes with enhanced expression in the pancreatic ducts were also similarly expressed in adenocarcinomas (Data S4).

Islet tumors were identified by a cluster of 311 genes with elevated expression (correlation ≥0.75). Consistent with their endocrine origin, these tumors were characterized by elevated levels of transcripts encoding chromogranins A and B (CHGA, CHGB), secretogranin II (SCG2), synaptophysin (SYP), peptidylglycine α-amidating monooxygenase (PAM), dopa decarboxylase (DDC), and somatostatin receptor 1 (SSTR1) (Data S5). Genes in the islet tumor cluster were generally expressed at high levels in the isolated islet cells but not in isolated pancreatic duct cells (Figure 2). Significance analysis identified genes differentially expressed in islet tumors compared to all other tissue samples in the dataset (Data S5). Two hundred genes were identified with a false discovery rate of 3.5%, of which 106 genes showed relatively high expression in the islet tumors. Consistent with previously published findings, genes with significantly elevated expression in islet cell tumors included insulinoma-associated 1 (INSM1), glutaminyl-peptide cyclotransferase (QPCT), fibrinogen B beta polypeptide (FGB), peroxisomal biogenesis factor 7 (PEX7). All of these genes identified by the SAM analysis were within the islet tumor cluster. Genes not previously described in islet cell tumors, but exhibiting elevated specific expression, include the AXL receptor tyrosine kinase (AXL) and the transcription factor V-maf musculoaponeurotic fibrosarcoma oncogene homolog B (MAFB). AXL expression is also elevated in leukemia, melanoma, prostate, colon, endometrial, and thyroid cancers[29]–[32]. Increased MAFB gene expression has been observed in myeloma [33]. INSM1, AXL, and MAFB are all located on chromosome 20 where comparative genomic hybridization studies have detected high-level amplification[34], [35].

A group of genes was expressed at higher levels in non-tumor and normal pancreatic tissues than in either islet cell tumors or adenocarcinomas. Acinar cells represent 90% of the cellular composition of the pancreas and are expected to dominate the expression profile of the normal pancreas. Genes encoding digestive enzymes produced by acinar cells such as amylase (AMY2A), elastase (ELA3A, ELA3B), carboxypeptidase A (CPA2), pancreatic lipase (PNLIP), and trypsin (PRSS2) were enriched in this cluster (Figures 1, “Acinar” cluster).

Genes known to be associated with the desmoplastic response in pancreatic cancer were clustered separately from the adenocarcinoma clusters. These genes included osteonectin (SPARC), fibronectin (FN1), versican (CSPG2), and collagen type 1A2 (COL1A2), tissue inhibitor of metalloproteinase 1 (TIMP1), biglycan (BGN), matrix metalloproteinase 2 (MMP2), and stromelysin-1 (MMP3) [5], [36]–[38]. The desmoplasia associated gene expression pattern was not seen in islet cell tumors, normal pancreatic tissues, pancreatic adenocarcinoma cell lines, or isolated pancreatic ducts, but was seen in other tumors such as lymphoma, sarcoma, osteoclast-like giant cell tumors, and adenocarcinomas. The expression of the stromal genes in tumors other than adenocarcinomas likely contributed to their segregation in a gene cluster distinct from the “Adeno” clusters, where expression was specifically associated with adenocarcinomas.

**Figure 4:**
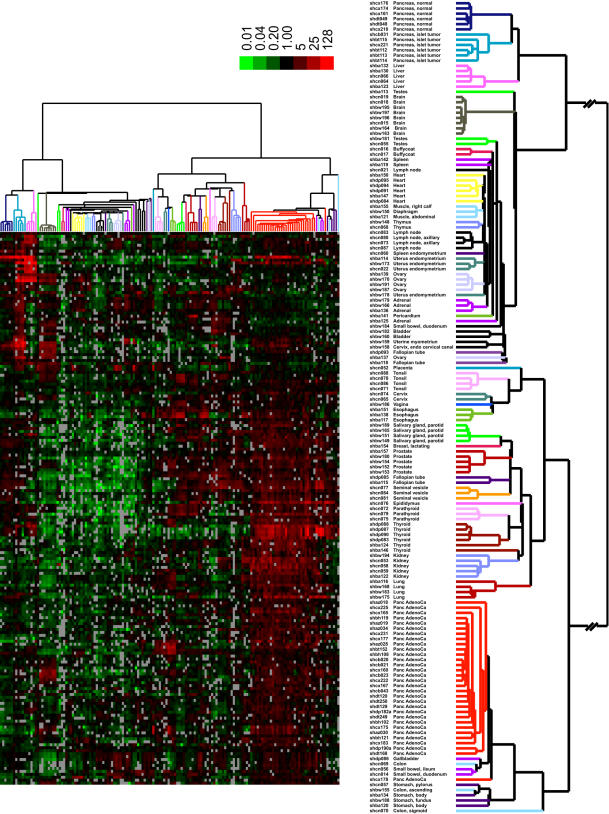
*Cluster analysis of secretory and membrane proteins.* Unsupervised hierarchical cluster analysis of expression of 187 genes encoding putative membrane or secreted proteins in normal pancreas, pancreatic adenocarcinoma, islet cell tumor tissues and a panel of normal tissues [9]. Only genes for which 80% of spots met quality criteria were used. The figure shows 187 genes representing secretory or membrane-bound proteins that show enhanced expression in pancreatic adenocarcinomas or islet cell tumors. An enlarged view of the array dendrogram along with sample identification is shown on the right. A color bar (*top*) relates color code to the magnitude of the differences in gene expression relative to the all-sample means for each gene. Grey indicates missing or excluded data.

Additional analysis was performed to identify genes potentially useful for diagnostic and therapeutic purposes. As secretory or membrane proteins may be especially useful as diagnostic markers or therapeutic targets, each gene enriched in pancreatic cancer or islet cell tumors was further analyzed using algorithms to identify signal peptide or transmembrane domains. For pancreatic cancer, only genes within the “Adeno” clusters were analyzed because they are enriched in adenocarcinoma cells and do not include the stromal genes common to other pancreatic diseases. Of the 259 genes in the “Adeno” clusters, 126 were classified as secretory or membrane proteins by amino acid sequence analysis. An additional 71 genes, for a total of 197, were identified using a recently defined dataset by Diehn et al. in which a genome-wide screen for secretory proteins was empirically determined using similar DNA microarrays [22]. For the 107 genes identified by significance analysis of microarrays with high expression in islet cell tumors, 32 were classified using sequence analysis as encoding secretory or membrane proteins, and an additional 20 genes were identified using the empirically derived dataset. These putative membrane/secreted protein encoding genes identified for pancreatic cancer and islet cell tumors were then examined using unsupervised hierarchical clustering in the context of a diverse collection of normal human tissues. The approach utilized the recently published gene expression profiles obtained from a diverse collection of normal human tissues, obtained using technologies and methods identical to those used in the present study [9]. The resultant set of putative secretory and membrane protein genes were able to distinctly cluster the pancreatic adenocarcinomas, islet cell tumors, and each of the normal tissues (Figure 4). None of the genes examined were uniquely expressed in pancreatic adenocarcinomas or islet cell tumors as each was detectably expressed in one or more normal tissues. Prediction analysis of microarrays (PAM), an algorithm that employs shrunken centroids [18] to identify genes capable of discriminating between different predetermined classes, identified a set of 16 genes whose expression pattern was capable of distinguishing 31 of 31 adenocarcinomas and a set of 12 genes whose expression pattern was capable of distinguishing 6 of 6 islet cell tumor samples from all normal tissues (Table 2 and Data S6). Four out of 121 non-adenocarcinomas were misclassified with the adenocarcinomas. None of the 150 non-islet cell tumor tissues were misclassified as tumors.

**Table 2:** Genes that discriminate adenocarcinoma and islet cell tumors from normal human tissues

Gene Symbol	Name	Clone ID
**Pancreatic Adenocarcinoma**		
LAMC2	Transcribed locus, homologous to laminin, gamma 2	IMAGE:460403
CTSE	Cathepsin E	IMAGE:243202
GPX2	Glutathione peroxidase 2 (gastrointestinal)	IMAGE:587847
LGALS4	Lectin, galactoside-binding, soluble, 4 (galectin 4)	IMAGE:511068
GPRC5A	G protein-coupled receptor, family C, group 5, member A	IMAGE:595037
MMP14	Matrix metallopeptidase 14 (membrane-inserted)	IMAGE:270505
ITGA2	Integrin, alpha 2 (CD49B, alpha 2 subunit of VLA-2 receptor	IMAGE:525246
AGR2	Anterior gradient 2 homolog (Xenopus laevis)	IMAGE:2321113
COL17A1	Collagen, type XVII, alpha 1	IMAGE:252259
TSPAN8	Tetraspanin 8	IMAGE:509731
GPRC5A	G protein-coupled receptor, family C, group 5, member A	IMAGE:1457276
CEACAM7	Carcinoembryonic antigen-related cell adhesion molecule 7	IMAGE:509688
**Islet Cell Tumors**		
SCG2	Secretogranin II (chromogranin C)	IMAGE:174627
RASSF7	Ras association (RalGDS/AF-6) domain family 7	IMAGE:1573778
TTR	Transthyretin (prealbumin, amyloidosis type I)	IMAGE:1868551
SGNE1	Neuroendocrine protein 1 (putative)	IMAGE:878836
INSM1	Insulinoma-associated 1	IMAGE:22895
PCSK2	Proprotein convertase subtilisin/kexin type 2	IMAGE:24254
QPCT	Glutaminyl-peptide cyclotransferase	IMAGE:711918
FGB	**Fibrinogen beta chain	IMAGE:84713
PEX7	Peroxisomal biogenesis factor 7	IMAGE:2018758
PTPRN2	Protein tyrosine phosphatase, receptor type	IMAGE: 812968
SERPINA1	Serpin peptidase inhibitor	IMAGE:294578
ARF3	ADP-ribosylation factor 3	IMAGE:291097

**Figure 5:**
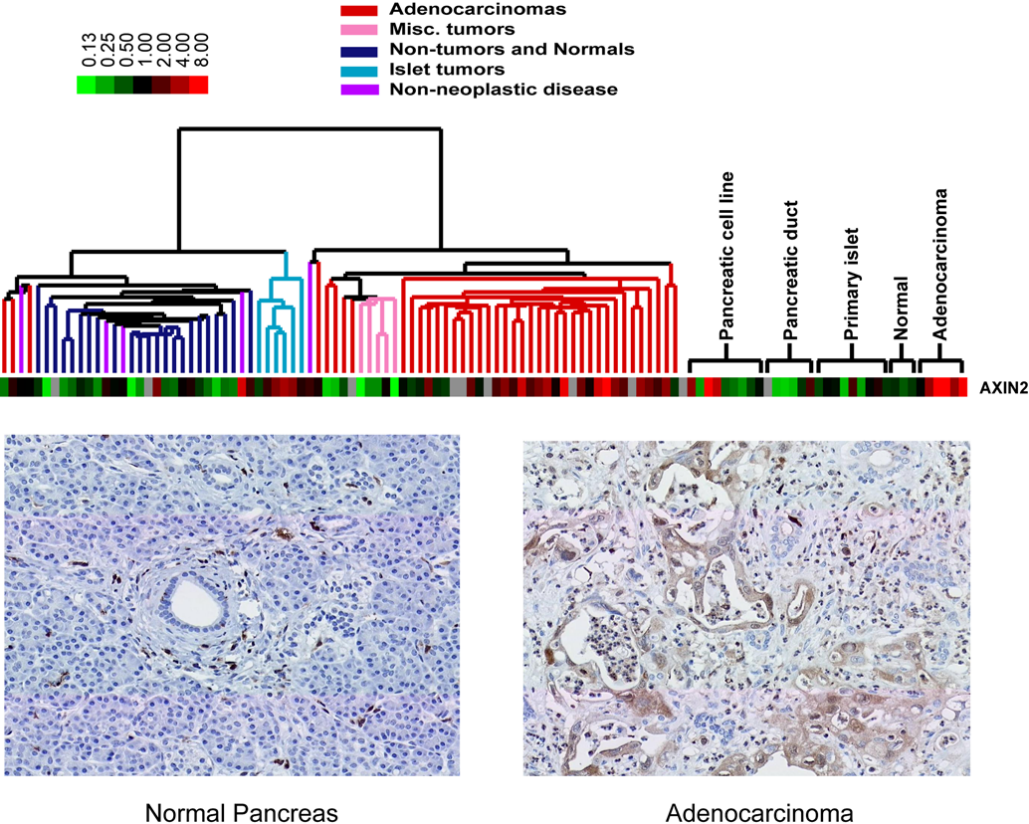
*Axin 2 expression in islet cell tumors and adenocarcinomas*. Pseudocolored image of axin 2 expression (top). Image represents mean centered values of sample/reference ratios. Representative immunohistochemistry with rabbit anti-axin2 antibodies in normal pancreas (bottom left) and pancreatic adenocarcinoma (bottom right).

Mutational activation of the Wnt pathway is one of the most common genetic steps in the pathogenesis of gastrointestinal cancers. Expression of AXIN2, a marker for *Wnt* pathway activation [39]–[41], was generally higher in pancreatic adenocarcinomas compared to primary pancreatic duct cells (Figure 5). AXIN2 expression was also elevated in islet cell tumors, which likely contributed to its clustering away from the previously identified adenocarcinoma gene clusters. Immunocytochemistry using tissue microarrays of pancreatic tissues revealed positive axin2 staining in 15 of 26 adenocarcinomas. No staining was seen in 13 normal pancreata.

## Discussion

DNA microarray analysis of global gene expression patterns in a diverse collection of normal and diseased pancreatic tissues allowed identification of specific gene expression profiles for pancreatic adenocarcinomas, islet cell tumors, and the normal pancreas. Interpretation of the gene expression profiles was facilitated by the parallel analysis of gene expression in primary pancreatic ducts and islets, and pancreatic cancer cell lines. The approach identified genes that are specifically expressed by neoplastic or stromal cells. Supporting the approach is the identification of many genes previously associated with pancreatic adenocarciomas or islet cell tumors. Many other genes not previously associated with these cancers were also newly identified. The availability of publicly available gene expression data provided an opportunity to compare the gene expression patterns of pancreatic adenocarcinomas and islet cell tumors to those of many other normal tissues. Other available datasets enabled the association of differentially expressed genes with areas of genomic DNA gains and losses. Similar gene expression datasets for other adenocarcinomas including hepatic, gastric, and breast tumors provide an opportunity for additional comparisons[9], [14], [42], [43]. In addition, the identification of secretory and membrane proteins using computational algorithms or annotations derived from genome-wide empirical assessments of mRNA localization further enhance of our ability to identify likely candidates for diagnostic and therapeutic purposes.

The present study also provides a characterization of the gene expression profile for islet cell tumors in the context of the whole pancreas. The gene expression profile for neuroendocrine tumors in patients with the MEN1 syndrome was recently reported [44]. The syndrome is characterized by neuroendocrine tumors of the parathyroids, pituitary, and pancreatic islets. Of the 6 patients with islet cell tumors studied, 2 exhibited insulinomas, which were compared to that of purified human islets from normal subjects. The differentially expressed genes in the MEN1 syndrome were not similar to those identified in this study, and suggest a different pathogenesis for spontaneous islet cell tumors. Our analysis identified additional genes, including AXL, MFAB, and AXIN2, whose elevated expression in islet cell tumors suggests signal transduction pathways important in their development.

Among pancreatic tumors, the adenocarcinomas are the most common and the most deadly. Distinct and characteristic differences in gene expression patterns have revealed subclasses of common tumors, such as breast cancer, that also differ with respect to their natural history or response to therapy[45]. In contrast, patients afflicted with pancreatic adenocarcinoma generally follow a similar course. Consistent with the uniformity of the clinical course, the gene expression profile for the majority of adenocarcinomas in the present study was quite uniform, suggesting that the disease is relatively homogenous. Although the present study is biased toward patients with surgically resectable tumors, differences in gene expression profiles would have been expected if significant biological differences among pancreatic adenocarcinomas were common.

Our results provide evidence that the Wnt pathway may often be activated in pancreatic adenocarcinoma and islet cell tumors. Evidence for Wnt pathway activation in pancreatic cancer has been inconclusive because nuclear staining for β-catenin, a mediator of Wnt signaling, is observed in less than 5% of cases [46]. Wnt pathway activation has been previously inferred from the altered cellular distribution of β-catenin in pancreatic adenocarcinomas, with decreased plasma membrane and increased cytoplasmic staining compared to normal pancreatic ducts and acinar cells by immunohistochemistry [46]. Recent studies have established that Axin2 expression is induced by Wnt pathway activation and serves as a useful marker. Higher expression of Axin2 in pancreatic adenocarcinomas and islet tumors compared to their non-neoplastic counterparts supports the potential importance of Wnt pathway activation in these tumors. A systematic search for mutations in genes involved in the Wnt pathway may yield new insights into the pathogenesis of these cancers.

The present study provides a comprehensive analysis of gene expression in pancreatic tumors. The analysis of a broad spectrum of pancreatic tissues in conjunction with publicly available datasets and software tools enhanced the identification of genes that may participate in disease pathogenesis, or may serve as preferred targets for diagnostic or therapeutic strategies.

## Supporting Information

Data S1 Clinical staging and outcomes for the samples used in Figure 1.
(0.03 MB XLS)

Data S2 List of genes within the Adeno 1 and 2 clusters. Annotated gene list identified by significance analysis of microarrays versus all other non-adenocarcinoma pancreatic tissues. Only the 31 adenocarcinomas grouped together in Figure 1 were used for the analysis. Worksheets also contain analysis for the identification of putative secretory and membrane proteins and genes that are associated with DNA copy gains and losses as determined by CGH. A table of contents is contained in the first Excel worksheet.
(1.91 MB XLS)

Data S3 Table of differentially expressed genes identified by SAM in adenocarcinomas whose genomic location showed high DNA copy gains or losses.
(0.03 MB XLS)

Data S4 List of genes differentially expressed between primary pancreatic duct cells and whole normal pancreatic tissue obtained using SAM as described in the Methods.
(2.12 MB XLS)

Data S5 List of genes within the islet cell tumor cluster. Annotated list of genes identified by SAM that are differentially expressed in islet tumors compared to all other pancreatic tissues in the dataset.
(0.43 MB XLS)

Data S6 Identification of candidate genes capable of discriminating pancreatic adenocarcinomas or islet cell tumors from a diverse collection of normal human tissues. Putative secretory or membrane proteins enriched in pancreatic adenocarcinomas or islet cell tumors in the current study were examined in the context of a wide variety of normal tissues reported in reference [9].
(0.05 MB XLS)
